# Effects of NH_4_CL application and removal on astrocytes and endothelial cells

**DOI:** 10.1186/s11658-016-0011-3

**Published:** 2016-08-23

**Authors:** Miha Bartolić, Andrej Vovk, Dušan Šuput

**Affiliations:** Institute of Pathophysiology, Faculty of Medicine, University of Ljubljana, Zaloška cesta 4, 1000 Ljubljana, Slovenia

**Keywords:** Hepatic encephalopathy, Hyperammonemia, Ammonia, Astrocytes, Endothelial cells, Volume, Calcium, pH

## Abstract

**Background:**

Hepatic encephalopathy (HE) is a complex disorder associated with increased ammonia levels in the brain. Although astrocytes are believed to be the principal cells affected in hyperammonemia (HA), endothelial cells (ECs) may also play an important role by contributing to the vasogenic effect of HA.

**Methods:**

Following acute application and removal of NH_4_Cl on astrocytes and endothelial cells, we analyzed pH changes, using fluorescence imaging with BCECF/AM, and changes in intracellular Ca^2+^ concentration ([Ca^2+^]_i_), employing fluorescence imaging with Fura-2/AM. Using confocal microscopy, changes in cell volume were observed accompanied by changes of [Ca^2+^]_i_ in astrocytes and ECs.

**Results:**

Exposure of astrocytes and ECs to 1 – 20 mM NH_4_Cl resulted in rapid concentration-dependent alkalinization of cytoplasm followed by slow recovery. Removal of the NH_4_Cl led to rapid concentration-dependent acidification, again followed by slow recovery. Following the application of NH_4_Cl, a transient, concentration-dependent rise in [Ca^2+^]_i_ in astrocytes was observed. This was due to the release of Ca^2+^ from intracellular stores, since the response was abolished by emptying intracellular stores with thapsigargin and ATP, and was still present in the Ca^2+^-free bathing solution. The removal of NH_4_Cl also led to a transient concentration-dependent rise in [Ca^2+^]_i_ that resulted from Ca^2+^ release from cytoplasmic proteins, since removing Ca^2+^ from the bathing solution and emptying intracellular Ca^2+^ stores did not eliminate the rise. Similar results were obtained from experiments on ECs. Following acute application and removal of NH_4_Cl no significant changes in astrocyte volume were detected; however, an increase of EC volume was observed after the administration of NH_4_Cl, and EC shrinkage was demonstrated after the acute removal of NH_4_Cl.

**Conclusions:**

This study reveals new data which may give a more complete insight into the mechanism of development and treatment of HE.

## Background

Hepatic encephalopathy (HE) comprises a broad spectrum of neurological and psychiatric dysfunctions seen in patients with liver disease. It can reflect diverse conditions, such as a reversible metabolic encephalopathy, brain atrophy, brain edema or a combination of them. Prevalent hypotheses suggest that a number of metabolic factors, present at the same time, are responsible for the development of HE. Neurotoxins, alteration of the blood-brain barrier (BBB), impairment of neurotransmission, altered brain energy metabolism and systemic response to infections and neuroinflammation are all factors playing a role in the pathogenesis of HE [1–3].

Ammonia is the most studied and well-described neurotoxin that precipitates HE [3–5], since an increase in brain ammonia level plays a critical role in its manifestation [5]. Although the correlation between venous ammonia levels and the severity of HE is still uncertain, lowering the blood ammonia concentration is the basis of present treatment of HE [6, 7].

Strong evidence suggests that astrocytes, which form the predominant cellular compartment in the brain [8], are the principal neural cells affected by ammonia toxicity [9]. Hyperammonemia (HA) is known to induce astrocyte swelling, which may be a key event in the development of HE [10]. HA, especially the acute elevation of ammonia level, leads to brain edema [11, 12]. Mechanisms underlying astrocyte swelling are not fully understood, although the metabolism of ammonia in astrocytes and the production of glutamine, which increases intracellular osmolarity, appear to be involved [13, 14]. Many studies have also shown that one of the earliest events in astrocytes exposed to ammonia is a rise in [Ca^2+^]_i_ [9, 13, 15, 16] that correlates with ammonia concentration [13]. Changes in astrocyte [Ca^2+^]_i_ may also play an important role modulating brain microcirculation [17]. However, little is known about how the removal of NH_4_Cl, as a treatment of HE, influences the affected cells [18].

Apart from affecting central nervous system cells directly, ammonia may also affect the blood–brain barrier (BBB) [19]. The associated endothelial cells (ECs) are the first cells in contact with blood containing ammonia during HA [20, 21]. Although the BBB is, structurally, made of endothelial cells connected by tight junctions and resting on the basal lamina, its function is far more complex, since it is influenced by interactions between endothelial cells, pericytes, smooth muscle cells and astrocytes and, occasionally, neuronal terminals [22]. The BBB is therefore difficult to study, and the effects of ammonia on endothelial cells, whether isolated or in contact with adjacent cells, have not been studied extensively. Data from the literature are controversial due to the use of different models and different NH_4_Cl concentrations. Hyperammonemia did not produce brain edema in experimental animals in vivo [23], but it caused profound swelling of brain slices exposed to ammonia [24]. Another phenomenon that has been suggested as the causative agent in the development of brain edema is change in the cerebral blood flow, resulting in altered intracranial blood volume [21, 25–27]. Both mechanisms are strongly influenced by the state of the capillary endothelial cells [20].

Changes of pH and [Ca^2+^]_i_ in astrocytes following the application of ammonia have been studied [13, 18], but there is no information on the acute effect of ammonia removal. The aim of the present study therefore was to further analyze pH and [Ca^2+^]_i_ changes after the acute removal of ammonia in order to give a more complete insight into the mechanisms of HE treatment. Additionally, the role of changes in EC morphology in the development of brain edema has been evaluated by analyzing changes in pH, [Ca^2+^]_i_ and volume in ECs following the addition and subsequent removal of ammonia.

## Methods

### Solutions

All solutions were prepared immediately prior to the experiment. The standard bathing solution (SBS) consisted of 150 mM NaCl, 5.4 mM KCl, 2 mM CaCl_2_, 1 mM MgCl_2_, 10 mM HEPES and 10 mM glucose; the pH was adjusted to 7.4 with NaOH. Ca^2+^-free solutions were prepared by substituting the CaCl_2_ with 2 mM EGTA as a calcium chelator. For the ammonia solutions, NH_4_Cl substituted the equivalent amount of NaCl in SBS in order to maintain the same osmolarity. Although the exact concentration of NH_4_^+^ in the cerebrospinal fluid of patients with HA is not known, NH_4_Cl levels in the arterial blood of patients with liver dysfunction reach roughly 1 mM and in patients with primary urea cycle disorders NH_4_Cl concentration can reach more extreme values [24], but values higher than 20 mM are improbable. In animal models of acute hyperammonemia, the NH_4_Cl concentration range was between 1.5 and 5 mM [23, 24]. To be able to compare our results to data from other authors [13, 25] we used 1 mM, 5 mM and 20 mM NH_4_Cl bathing solutions.

### Materials

Fluorophores Fura-2/AM, BCECF/AM and FM 1-43FX were obtained from Molecular Probes, Inc., USA. Minimum Essential Medium Eagle (MEM Eagle), Dulbecco’s Modified Eagle Medium/Nutrient Mixture F-12 (DMEM/F12), fetal bovine serum (FBS) and N-2-hydroxyethylpiperazine-N-2-ethane sulfonic acid (HEPES) were from GIBCO, Invitrogen Corp, USA. ATP, thapsigargin, penicillin and streptomycin were from Sigma-Aldrich Co.

### Astrocyte cultures

Animals were kept on a 12 h light-dark cycle in a temperature-controlled colony room at 22–24 °C with free access to rodent pellets and tap water. They were handled according to the NIH Guide for the Care and Use of Laboratory Animals, and all experiments were carried out in accordance with the European Council Directive of November 24th, 1986 (86/609/EEC) and Slovenian legislation. The number of animals was kept at a minimum by preparing primary cultures in a central laboratory for researchers working on astrocytes and neurons. For our experiments astrocyte cultures were prepared from the cerebral cortex of 36 animals. For each series of experiments the cells from three Wistar Hannover rats were pooled. A slightly different method from that described by Čarman-Kržan [28] was used. 1–3 day old rats were anesthetized, decapitated and the brains removed aseptically. The meninges were removed and the tissue dissociated by passage through sterile Nitex nylon screens (75 μm mesh size) into 10 ml of culture medium composed of DMEM/F12 (1:1), 10 % (vol/vol) FBS, 100 U/ml penicillin, and 100 μg/ml streptomycin. The cell suspension was diluted and plated into 75 cm^2^ tissue-culture flasks. Cells were grown at 37 °C in a water-saturated air environment containing 5 % CO_2_ until confluence. The culture medium was changed every three days. The cell cultures were shaken at 200 rpm overnight and the culture medium changed the following day. The procedure was repeated 3 times to remove small process-bearing cells on the protoplasmic cell layer (mainly oligodendrocyte progenitors and microglia). The cell cultures were trypsinized and plated onto 100-mm culture dishes and further cultured for 7–10 days. Under these conditions, astroglial-enriched cultures were obtained (95–98 % of cells showed immunoreactivity for glial fibrillary acidic protein). Analyses were carried out on astrocytes plated on poly-L-lysine-coated coverslips at a concentration of 50 000 cells per ml that had been grown for 5 days until reaching a sub-confluent state [29].

### Endothelial cell cultures

We used the endothelial cell line T24 (ECs) derived from human bladder. Cells were grown in cell flasks in cell medium at 37 °C in an environment of water-saturated air/5 % CO_2_. Every 3 – 4 days (depending on cell density and state of cell confluence) the cell medium was removed and the cell cultures were trypsinized and again plated in cell flasks with cell medium under the same conditions. For experimental purposes ECs were plated on a coverslip.

### Measurement of intracellular H^+^ and intracellular Ca^2+^ concentrations

Fluorescence imaging was performed using an Axiovert S100 microscope (Zeiss) with Fluar 10x/0.5 and Fluar 40x/1.3 oil objectives combined with a Polychromator II (T.I.L.L. Photonics GmbH, Germany) light source and Coolsnap HQ2 camera (Photometrics). The Visiview 2.0.1 (Visitron Systems GmbH, Germany) computer software was used to acquire images and to measure intensities at different wavelengths. Before the beginning of any experiment the viability of cells on coverslips was checked under a light microscope. The cells were incubated in 0.5 μM BCECF/AM in SBS at room temperature (21 °C) in the dark for 15 – 20 min, then rinsed with SBS and again incubated in SBS for 10 – 15 min for complete de-esterification of intracellular AM esters. Upon completion of labeling, the coverslips with the cells were placed on a coverslip holder on the microscope. A group of cells without visible deformities was located under a light microscope. These cells were studied with microspectrofluorometry, using excitation wavelengths of 490 nm and 440 nm; emitted radiation was passed through a filter that passes wavelengths higher than 530 nm. The images acquired at the two excitation wavelengths were stored at intervals of 5 s to capture the rapid changes during the first minute after application or removal of NH_4_Cl and then at intervals of 60 s for another 9 min. Each cell was outlined manually and its fluorescence intensity under excitation at 490 and 440 nm measured. The background fluorescence intensity was subtracted, and an intensity ratio for each cell calculated. The ratio of fluorescence intensities at 490 nm to that at 440 nm (B_490_/B_440_) was used to determine [H^+^]_i_. During experiments SBS was exchanged as appropriate for a solution with a different concentration of ammonia which, after 10 min, was again changed for SBS [13].

To determine [Ca^2+^]_i,_ astrocytes were labeled with Fura-2/AM at 4 μM. The protocol was similar to that for [H^+^]_i_ measurement, except that excitation wavelengths of 340 nm (F_340_) and 380 nm (F_380_) and a filter passing wavelengths higher than 510 nm were used [13]. The ratio (F_340_/F_380_) was used to determine [Ca^2+^]_i_.

### Measurement of cell volume

The imaging system for volume analysis comprised a laser confocal microscope Leica TCS SP (Leica, Heidelberg, Germany) with HC PL Fluotar 20x0.5 and PL APO Fluotar 100x1.4 oil objectives and Leica Confocal Software 2.0 (Leica Microsystems Heidelberg GmbH, Germany). Bitplane Imaris 7.4.2 software (Bitplane AG, Switzerland) was used for image analysis. Before the beginning of each experiment, the viability of cells on coverslips was checked under a light microscope. The cells were incubated in 5 μM membrane stain FM 1-43FX at room temperature (21 °C) in the dark for 15 min on the microscope holder and then rinsed with SBS. A group of cells without visible deformities was first identified under a light microscope. 3D images of cells were acquired using confocal microscopy with an excitation wavelength of 488 nm (He/Ne laser). Acquired spectra between 555 nm and 585 nm were used. Series of 32 slices at 100x magnification, with a resolution of 512 x 512, were recorded. Using the Bitplane Imaris 7.4.2 computer software, 3D objects were created from the acquired images and the volume of each cell measured, based on object-containing voxels, at specific time points during the experiment. Relative volume changes were expressed as % change of the original volume.

### Measurement of cell areas

Images of those cells acquired during the experiments while measuring [H^+^]_i_ were further analyzed with the ImageJ (National Institutes of Health) computer software for measuring cell areas. The contrast of the images was enhanced to better determine cell borders. Each visible cell was then outlined at the beginning of the experiment, before and after each solution exchange and at the end of the experiment. Changes of areas were then calculated.

### Statistical methods

Analysis of 101 experiments dealing with changes of fluorescence ratio, area and volume before and after the addition of NH_4_Cl and before and after its removal showed identical directions of changes. The directions of changes are presented as trend plots. We performed a nonparametric Wilcoxon signed rank test for each experiment, to test the hypothesis that there is no change in values of fluorescence ratio recorded before and after the addition of NH_4_Cl, and also before and after the removal of NH_4_Cl. Group analysis was then performed by combining the rank sum tests of all experiments [30]. Weighted means (and pooled SDs) were also calculated for all experiments to assess the average relative change of fluorescence ratio values after each type of treatment. Cell volume and area changes after the application and after the removal of NH_4_Cl were analyzed using the same statistical approach.

*N* is the number of experiments in one group of experiments (one coverslip = one experiment) and *n* is the total number of cells studied. All statistical analyses were performed using R computer software. Changes were considered significant at *p* < 0.01. All numerical results in the text are expressed as weighted means ± pooled standard deviation.

## Results and discussion

### NH_4_Cl triggers intracellular pH changes in astrocytes

**Fig. 1:**
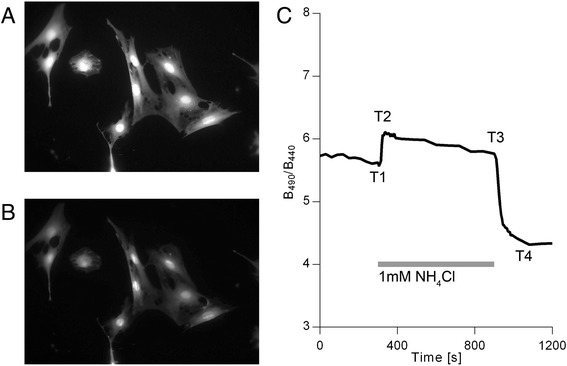
NH_4_Cl triggers intracellular pH changes in astrocytes. **a** and **b** – Fluorescence images, acquired using an excitation wavelength of 490 nm, of a group of astrocytes loaded with BCECF/AM. **a** – Astrocytes at the beginning of the experiment. **b** – The same cells after being exposed to NH_4_Cl. The morphology of the cells remained unchanged. **c** – An example of average B_490_/B_440_ as a function of time in astrocyte cell culture (n = 10). Application of 1 mM NH_4_Cl caused a rapid rise of B_490_/B_440_ followed by a slow decline. Removal of the NH_4_Cl by substituting it with SBS caused a rapid fall of B_490_/B_440._ T1 – time point before the substitution of the SBS with the NH_4_Cl bathing solution; T2 – time point at which the maximum change of B_490_/B_440_ was reached after the substitution of the SBS with the NH_4_Cl bathing solution; T3 – time point (at 900 s) before substituting the NH_4_Cl bathing solution with the SBS; T4 – time point of the maximum change of B_490_/B_440_ after substituting the NH_4_Cl bathing solution with the SBS

Extracellular application of NH_4_Cl triggered a rapid rise in B_490_/B_440_ (Fig. 1c). This can be explained by a rapid influx of NH_3_, consuming intracellular H^+^ for NH_4_^+^ formation, thereby increasing the intracellular pH (pH_i_). After the initial increase a slow decline in B_490_/B_440_ was observed. This recovery of pH_i_ is a consequence of NH_4_^+^ continuing to enter the cells after the NH_3_/NH_4_^+^ equilibrium has been reached, driven by the concentration gradient and membrane potential [31]. After incubation for 10 min in the NH_4_Cl solution, the latter was rapidly exchanged for SBS. The removal of NH_4_Cl resulted in a rapid decrease in B_490_/B_440_, again followed by a slow rise (Fig. 1c). The changes observed after the acute fall of extracellular ammonia level are the result of reversal of the process described above. During these experiments the morphology of the astrocytes remained intact (Fig. 1a and b).

**Fig. 2:**
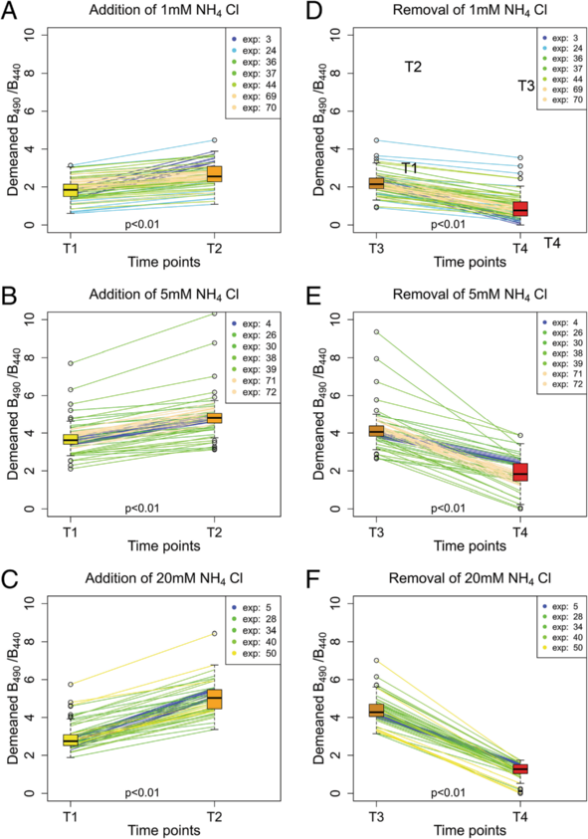
NH_4_Cl triggers intracellular pH changes in astrocytes. **a**, **b** and **c** – Changes after addition of 1 mM, 5 mM and 20 mM NH_4_Cl plotted as trends. **d**, **e** and **f** – Changes after removal of 1 mM, 5 mM and 20 mM NH_4_Cl plotted as trends; boxplots on each side present median, upper and lower quartile, minimum and maximum and outliers. Experiments are numbered using consecutive numbers as performed. T1 – time point before the substitution of the SBS with the NH_4_Cl bathing solution; T2 – time point at which the maximum change of B_490_/B_440_ was reached after the substitution of the SBS with the NH_4_Cl bathing solution; T3 – time point (at 900 s) before substituting the NH_4_Cl bathing solution with the SBS; T4 – time point of the maximum change of B_490_/B_440_ after substituting the NH_4_Cl bathing solution with the SBS. Experiments are numbered using consecutive numbers as performed

The relative increase of B_490_/B_440_ after adding 1 mM NH_4_Cl was 15.2 % ± 2.4 % (*p* < 0.01; *N* = 7; *n* = 80). Addition of 5 mM and 20 mM NH_4_Cl triggered greater increases of 20.1 % ± 2.0 % (*p* < 0.01; *N* = 7; *n* = 79) and 46.3 % ± 6.1 % (*p* < 0.01; *N* = 5; *n* = 60) (Fig. 2a, b and c). Resubstituting the extracellular solutions of 1 mM, 5 mM and 20 mM NH_4_Cl with the standard bathing solution resulted in a relative decrease of B_490_/B_440_ of 21.9 % ± 2.5 % (*p* < 0.01; *N* = 7; *n* = 80), 35.9 % ± 2.0 % (*p* < 0.01; *N* = 7; *n* = 79) and 51.6 % ± 2.6 % (*p* < 0.01; *N* = 5; *n* = 60) (Fig. 2d, e and f).

The results clearly show that the exposure of cells to NH_4_Cl leads to a rapid increase of pH_i_ followed by a slow decline and that the removal of extracellular NH_4_Cl leads to a rapid pH_i_ fall and a slow recovery. All pH_i_ changes correlate with the extracellular NH_4_Cl concentration. The results are consistent with the previously described mechanism of intracellular pH_i_ shifts with NH_4_Cl [31, 32].

### Addition and removal of NH_4_Cl stimulates changes of [Ca^2+^]_i_ in astrocytes

**Fig. 3:**
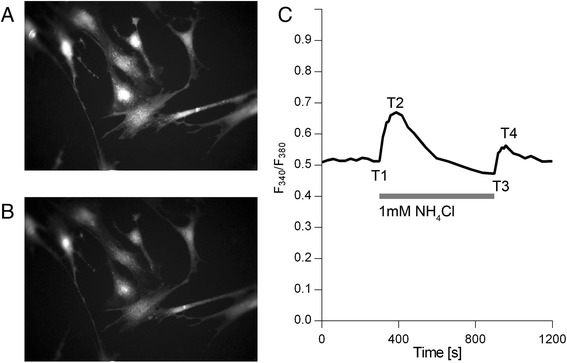
NH_4_Cl addition and removal stimulates [Ca^2+^]_i_ changes in astrocytes. **a** and **b** – Fluorescence images, acquired using an excitation wavelength of 380 nm, of a group of astrocytes loaded with Fura-2/AM. A – Astrocytes at the beginning of the experiment. **b** – The same cells after being exposed to NH_4_Cl. The morphology of the cells remained unchanged. **c** – An example of average F_340_/F_380_ as a function of time in astrocyte cell culture (n = 7). T1 – time point before the substitution of the SBS with the NH_4_Cl bathing solution; T2 – time point at which the maximum change of F_340_/F_380_ was reached after the substitution of the SBS with the NH_4_Cl bathing solution; T3 – time point (at 900 s) before substituting the NH_4_Cl bathing solution with the SBS; T4 – time point of the maximum change of F_340_/F_380_ after substituting the NH_4_Cl bathing solution with the SBS

**Fig. 4:**
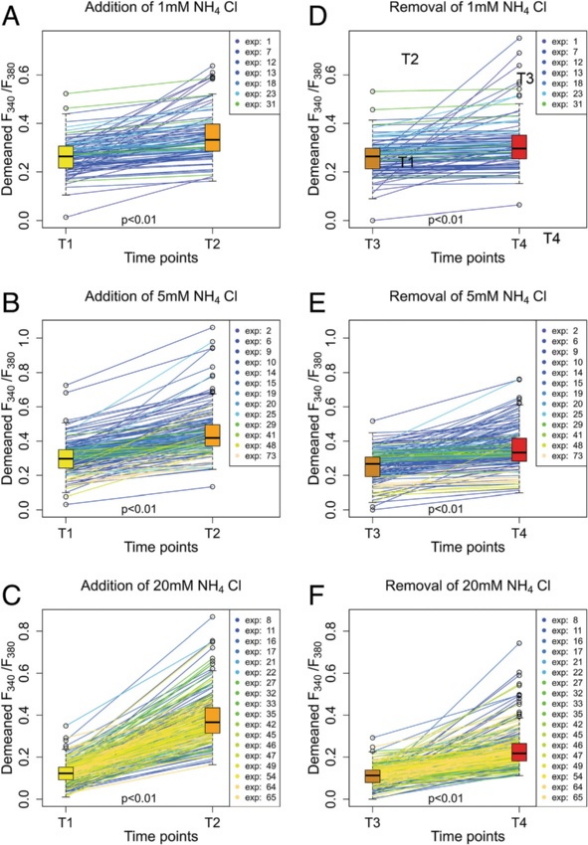
NH_4_Cl addition and removal stimulate [Ca^2+^]_i_ changes in astrocytes. **a**, **b** and **c** – Changes after addition of 1 mM, 5 mM and 20 mM NH_4_Cl plotted as trends. **d**, **e** and **f** – Changes after removal of 1 mM, 5 mM and 20 mM NH_4_Cl plotted as trends; boxplots on each side present median, upper and lower quartile, minimum and maximum and outliers. T1 – time point before the substitution of the SBS with the NH_4_Cl bathing solution; T2 – time point at which the maximum change of F_340_/F_380_ was reached after the substitution of the SBS with the NH_4_Cl bathing solution; T3 – time point (at 900 s) before substituting the NH_4_Cl bathing solution with the SBS; T4 – time point of the maximum change of F_340_/F_380_ after substituting the NH_4_Cl bathing solution with the SBS. Experiments are numbered using consecutive numbers as performed

Changes of [Ca^2+^]_i_ were observed using the calcium indicator Fura-2/AM. Given that a large proportion of the intracellular Ca^2+^ is bound to cytoplasmic proteins, an increase in intracellular pH caused by the addition of NH_4_Cl should result in a decrease of [Ca^2+^]_i_. The reduction in [H^+^]_i_ results in the release of H^+^ from cytoplasmic proteins; thus free Ca^2+^ ions fill up the freed protein-binding sites [33]. This mechanism suggests that a rise in pH_i_ should lead to a fall of [Ca^2+^]_i_. However our results, as well as those of previous studies [13, 15, 16], show that [Ca^2+^]_i_ rises after acute alkalization of cytoplasm by NH_4_Cl (Fig. 3c) and that higher concentrations of NH_4_Cl elicit greater increases of [Ca^2+^]_i_. In our experiments the addition of 1 mM NH_4_Cl resulted in a 18.3 % ± 12.0 % (*p* < 0.01; *N* = 7; *n* = 93) increase of F_340_/F_380_ and the addition of 5 mM and 20 mM NH_4_Cl resulted in 28.1 % ± 16.6 % (*p* < 0.01; *N* = 13; *n* = 171) and 59.5 % ± 17.2 % (*p* < 0.01; *N* = 18; *n* = 266) increases respectively (Fig. 4a, b and c).

Since the rise in [Ca^2+^]_i_ following a rise of pH_i_ cannot be explained by an increase of binding sites on cytoplasmic proteins, it must be due either to the influx of Ca^2+^ through the cell membrane or to its release from intracellular storage [13]. To check the possibility of Ca^2+^ entry into the cells, changes in [Ca^2+^]_i_ were compared following the exposure of astrocytes to NH_4_Cl in SBS and to astrocytes in the Ca^2+^-free bathing solution. No statistically significant differences were observed (*p* = 0.21), suggesting that intracellular Ca^2+^ stores must be responsible for the rapid increase in [Ca^2+^]_i_ following exposure to NH_4_Cl. In a Ca^2+^-free bathing solution, exposure to 20 mM NH_4_Cl resulted in an increase of F_340_/F_380_ of 54.6 % ± 19.6 % (*p* < 0.01; *N* = 4; *n* = 46).

The suggestion that Ca^2+^ is released from intracellular stores was further tested by depleting the intracellular stores. Thapsigargin (500 nM), which blocks Ca^2+^ transport into the endoplasmic reticulum (ER) and prevents the refilling of calcium stores, and ATP (100 μM), which stimulates Ca^2+^ release from ER stores, were added [13] to the Ca^2+^-free bathing solution before the start of the experiment. Addition of 20 mM NH_4_Cl to the astrocytes pretreated with thapsigargin and ATP resulted in a decrease in F_340_/F_380_ of 26.5 % ± 8.5 % (*p* < 0.01, *N* = 12; *n* = 88). After intracellular Ca^2+^ stores were depleted, the fall in [H^+^]_i_ following the addition of NH_4_Cl resulted in release of H^+^ from cytoplasmic proteins. The newly freed protein-binding sites were filled by intracellular Ca^2+^, resulting in a reduction in [Ca^2+^]_i_.

The release of Ca^2+^ from intracellular stores demonstrated after alkalinization of astrocytes by NH_4_Cl is consistent with reported results [13]. Further experiments were therefore performed to determine how the removal of extracellular NH_4_Cl and the acidification of astrocytes influence the intracellular Ca^2+^.

After the astrocytes were incubated for 10 min in a solution containing NH_4_Cl, the solution was rapidly substituted by SBS, at the same time measuring F_340_/F_380_. A temporary rise in F_340_/F_380_ was observed that was lower than the rise of F_340_/F_380_ seen during the addition of NH_4_Cl (Figs. 3c and 4).

The exchange of the 1 mM NH_4_Cl solution with SBS resulted in a rise in F_340_/F_380_ of 12.3 % ± 19.5 % (*p* < 0.01; *N* = 7; *n* = 93). The exchange of bathing solutions of 5 mM and 20 mM NH_4_Cl resulted in rises of 20.4 % ± 16.5 % (*p* < 0.01; *N* = 13; *n* = 171) and 30.4 % ± 19.1 % (*p* < 0.01; *N* = 18; *n* = 266) respectively (Fig. 4d, e and f). The fall of pH always preceded the increase of intracellular Ca^2+^. These results show that acidification of astrocytes leads to a transient rise in [Ca^2+^]_i_, the magnitude being dependent on the concentration of NH_4_Cl in the bathing solution before its washout. This is similar to the results obtained on cultured astrocytes caused by alkalinization and acidification using the weak base trimethylamine [13]. The same authors have shown that, in a different experimental setup using astrocytes within brain slices, alkalinization and acidification by the application and washout of 10 mM trimethylamine does not cause any changes in intracellular Ca^2+^ [18]. The difference in the results may, at least in part, be due to the use of brain slices and not isolated astrocytes. Evidently the study on isolated cells is only a minor step in understanding the complex interactions between cells within the nervous system.

To determine the source of the increase of Ca^2+^ described above, changes of F_340_/F_380_ were also recorded following the removal of NH_4_Cl in the experiments in the bathing solution without Ca^2+^, and in the experiments on cells pretreated with the combination of thapsigargin and ATP in Ca^2+^-free solution.

A transient rise in F_340_/F_380_ (47.9 % ± 10.0 % (*p* < 0.01; *N* = 4; *n* = 46) was observed, even in the Ca^2+^-free bathing solution, suggesting that extracellular Ca^2+^ does not play a significant role in the detected rise of [Ca^2+^]_i_. Even after depleting intracellular stores with thapsigargin and ATP, the removal of NH_4_Cl led to an increase in F_340_/F_380_ of 79.3 % ± 27.6 % (*p* < 0.01; *N* = 12; *n* = 88). The [Ca^2+^]_i_ rise is probably a result of the release of Ca^2+^ from cytoplasmic proteins.

### Changes in astrocyte volume on addition and removal of NH_4_Cl

After determining the changes in H^+^ and Ca^2+^ concentrations in astrocytes that occurred on substituting SBS with 20 mM NH_4_Cl, then resubstituting the bathing solution back with SBS, changes in cell volume following these acute events were investigated. Volume changes immediately and approximately 10 min after acute exchange of the bathing solutions were determined by confocal microscopy. However, no significant changes in astrocyte volume during the experiment were detected, which is consistent with other studies, in which a progressive increase of the cell volume of astrocytes exposed to NH_4_Cl was observed only after 1–3 days [34]. Our data are also in agreement with the observation that hyperammonemia does not produce brain edema in vivo [23].

### NH_4_Cl triggers intracellular pH changes in endothelial cells

Endothelial cells are an essential part of the blood brain barrier, and their function is regulated by astrocytes as well as by metabolites, cytokines and other biologically active molecules [22, 35]. These endothelial cells are involved in the regulation of transport of molecules between the blood and the cerebrospinal fluid, and their function appears to serve specific roles within the nervous system. Endothelial cells in the brain are difficult to study due to their small size and their complex interaction with other cells such as pericytes, smooth muscle cells and astrocytes. Primary endothelial cells from other sources and endothelial cell lines differ from those in the brain; therefore the results obtained on endothelial cell lines must be interpreted cautiously.

**Fig. 5:**
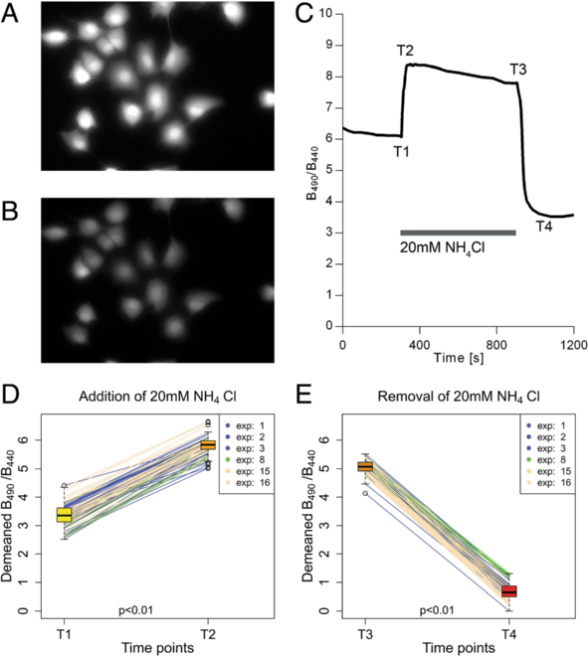
NH_4_Cl triggers intracellular pH changes in endothelial cells. **a** and **b** – Fluorescence images, acquired using an excitation wavelength of 490 nm, of a group of ECs loaded with BCECF/AM. A – ECs at the beginning of the experiment. **b** – The same cells after being exposed to NH_4_Cl. The morphology of the cells remained unchanged. **c** – An example of average B_490_/B_440_ as a function of time in EC cell culture (*n* = 18). T1 – time point before the substitution of the SBS with the NH_4_Cl bathing solution; T2 – time point at which the maximum change of B_490_/B_440_ was reached after the substitution of the SBS with the NH_4_Cl bathing solution; T3 – time point (at 900 s) before substituting the NH_4_Cl bathing solution with the SBS; T4 – time point of the maximum change of B_490_/B_440_ after substituting the NH_4_Cl bathing solution with the SBS; **d** – Changes after NH_4_Cl addition plotted as trends. **e** – Changes after removal of NH_4_Cl plotted as trends. Boxplots on each side present median, upper and lower quartile, minimum and maximum and outliers. Experiments are numbered using consecutive numbers as performed

Experiments for measuring intracellular H^+^ changes were repeated here with ECs, and similar results to those from experiments with astrocytes were found. Extracellular application of NH_4_Cl triggered a rapid rise in B_490_/B_440_, after which a slow decline in B_490_/B_440_ was observed. After 10-min incubation with the NH_4_Cl solution, the solution was rapidly exchanged with SBS. The removal of NH_4_Cl resulted in a rapid decrease in B_490_/B_440_, again followed by a slow rise, due to the same mechanism as in experiments with astrocytes (Fig. 5c). An increase in B_490_/B_440_ of 41.8 % ± 6.4 % (*p* < 0.01; *N* = 6; *n* = 89) after exposure to 20 mM NH_4_Cl and a decrease of 56.2 % ± 1.6 % (*p* < 0.01; *N* = 6; *n* = 89) after removal of NH_4_Cl were observed (Fig. 5d and e).

### Addition and removal of NH_4_Cl stimulates changes in [Ca^2+^]_i_ in endothelial cells

**Fig. 6:**
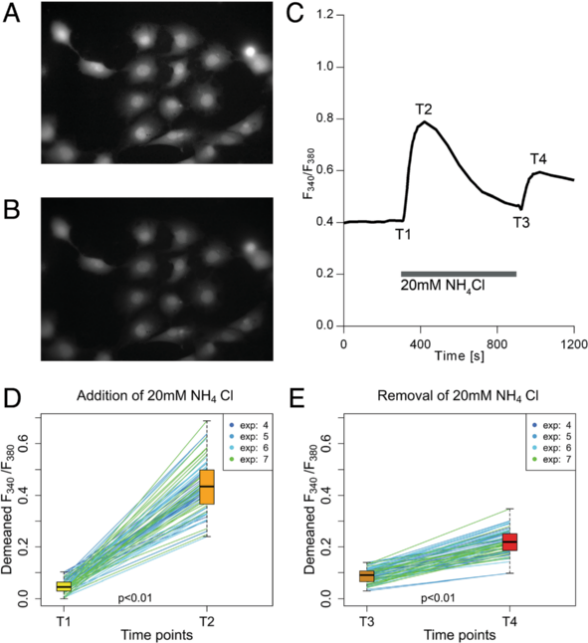
NH_4_Cl addition and removal stimulates [Ca^2+^]_i_ changes in endothelial cells. **a** and **b** – Fluorescence images, acquired using an excitation wavelength of 380 nm, of a group of ECs loaded with Fura-2/AM. A – ECs at the beginning of the experiment. **b** – The same cells after being exposed to NH_4_Cl. The morphology of the cells remained unchanged. **c** – An example of average F_340_/F_380_ as a function of time in astrocyte cell culture (*n* = 18). T1 – time point before the substitution of the SBS with the NH_4_Cl bathing solution; T2 – time point at which the maximum change of F_340_/F_380_ was reached after the substitution of the SBS with the NH_4_Cl bathing solution; T3 – time point (at 900 s) before substituting the NH_4_Cl bathing solution with the SBS; T4 – time point of the maximum change of F_340_/F_380_ after substituting the NH_4_Cl bathing solution with the SBS; **d** – Changes after NH_4_Cl addition plotted as trends. **e** – Changes after NH_4_Cl removal plotted as trends. Boxplots on each side present median, upper and lower quartile, minimum and maximum and outliers. Experiments are numbered using consecutive numbers as performed

Changes of [Ca^2+^]_i_ in ECs were observed using the same protocol as in the experiments with astrocytes. In the SBS both addition and removal of NH_4_Cl resulted in an increase in F_340_/F_380_ followed by a slow decline (Fig. 6c). However, the first peak of F_340_/F_380_ following the addition of 20 mM NH_4_Cl was much higher (99.8 % ± 27.6 % *p* < 0.01; *N* = 4; *n* = 72) (Fig. 6d) than that after the removal of NH_4_Cl (30.2 % ± 8.3 %; *p* < 0.01; *N* = 4; *n* = 72) (Fig. 6e). Similar results were also expected for the Ca^2+^-free bathing solution, but the increase in [Ca^2+^]_i_ after the addition of NH_4_Cl was much smaller (34.0 % ± 16.0 %; *p* < 0.01; *N* = 2; *n* = 29). Such a difference was not detected in astrocytes. This could suggest that NH_4_Cl stimulates not only release of Ca^2+^ from intracellular stores but also an influx of extracellular Ca^2+^ into ECs. However, the efflux of Ca^2+^ from cells in the Ca^2+^-free bathing solution [36], which could be expressed differently in ECs and in astrocytes, must be taken into consideration. The removal of NH_4_Cl in the Ca^2+^-free bathing solution resulted in an increase of F_340_/F_380_ of 40.9 % ± 10.2 % (*p* < 0. 05; *N* = 2; *n* = 29).

Intracellular Ca^2+^ stores were further depleted by the addition of thapsigargin to a final concentration of 500 nM and ATP to a final concentration of 100 μM [13] before measuring changes of [Ca^2+^]_i_. After the addition of NH_4_Cl, a small increase in F_340_/F_380_ (3.9 % ± 2.9 %; *p* < 0.01; *N* = 4; *n* = 73) was observed but, after its removal, a rise in F_340_/F_380_ (43.0 % ± 17.0 %; *p* < 0.01; *N* = 4; *n* = 73) similar to that observed in ECs in Ca^2+^-free solution without intracellular Ca^2+^ stores being depleted (*p* = 0.092) was measured.

Our results suggest that the addition of NH_4_Cl to ECs triggers a transient increase in [Ca^2+^]_i_ that is attenuated in ECs in Ca^2+^-free bathing solution and almost abolished in ECs after the depletion of intracellular Ca^2+^ stores in the Ca^2+^-free bathing solution. However, acute removal of NH_4_Cl from the extracellular solution provoked a transient rise in [Ca^2+^]_i_ which could not be abolished, either by substituting the SBS with Ca^2+^-free bathing solution or by further depleting intracellular Ca^2+^ stores, suggesting that the increase in [Ca^2+^]_i_ is due to a release of Ca^2+^ from cytoplasmic proteins.

### The volume of ECs changes following the addition and subsequent removal of NH_4_Cl

Endothelial cells may play a major role in vasogenic mechanisms of HE. It has been shown that brain slices swell after exposure to 10 mM or higher concentrations of NH_4_Cl [24]. In our experiments astrocyte volume did not change significantly even after exposure to 20 mM NH_4_Cl. Therefore experiments on ECs were performed to test the hypothesis that ammonia triggers volume changes in ECs. EC volume changes could result in brain blood flow changes and consequently in changes of intracerebral blood volume or cerebral edema [21, 25–27].

Volume changes of ECs were measured after the administration and subsequent removal of NH_4_Cl, using confocal microscopy and computer software to calculate maximum volume changes. The time points at which the solutions were exchanged were the same as in the previous experiments when pH and [Ca^2+^]_i_ were measured. Exposure of EC to different solutions was relatively short, and it is not possible to exclude further volume changes of ECs that might have appeared after a longer exposure to NH_4_Cl.

**Fig. 7:**
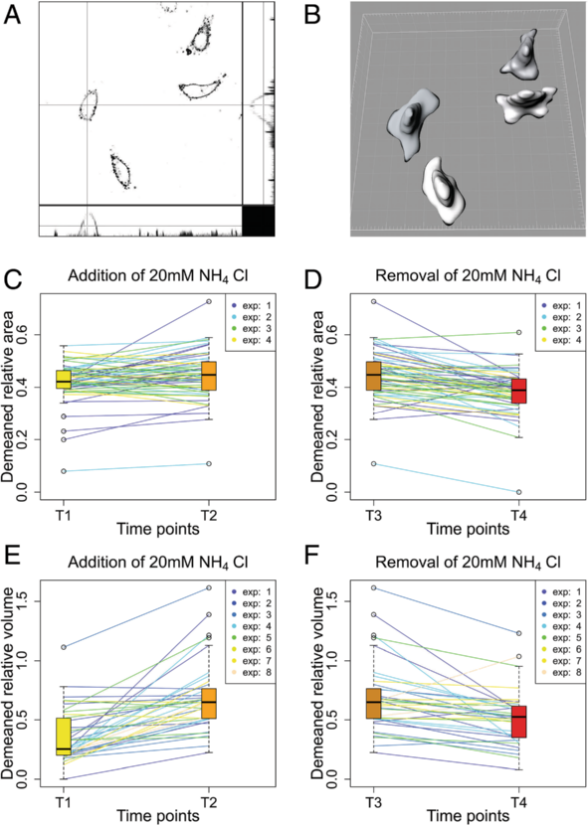
Changes in volume of ECs following the addition and subsequent removal of NH_4_Cl. **a** – A group of ECs observed under confocal microscopy and presented in Imaris Section View in three perspectives. **b** – The same group of ECs presented as 3D objects. The volume for each cell was calculated based on voxel count. **c** – Relative area changes after NH_4_Cl addition plotted as trends. **d** – Relative area changes after removal of NH_4_Cl plotted as trends. **e** – Relative volume changes after NH_4_Cl addition plotted as trends. **f** – Relative volume changes after NH_4_Cl removal plotted as trends. Experiments are numbered using consecutive numbers as performed

Exchanging the SBS with 20 mM NH_4_Cl (Fig. 7e) resulted in an average increase of EC volume of 35.1 % ± 27.6 % (*N* = 8; *n* = 37; *p* < 0.01), and returning to SBS resulted in a markedly smaller cell shrinkage of 11.0 % ± 13.0 % (*N* = 8; *n* = 37; *p* < 0.01) (Fig. 7f). While our experiments were of relatively short duration – or at least too short to detect astrocyte volume changes [34] – the volume changes in ECs were demonstrated 10 min after the bathing solution exchange. To obtain a better insight into the changes in cell morphology, EC surface projected areas were also measured. An EC area expansion of 2.6 % ± 6.4 % (*N* = 4; *n* = 59; *p* < 0.01) was recorded after changing SBS for 20 mM NH_4_Cl (Fig. 7c) and an area shrinkage of 6.2 % ± 6.9 % (*N* = 4, *n* = 59; *p* < 0.01) after substituting 20 mM NH_4_Cl with SBS (Fig. 7d). The results show that the volume increase of ECs is greater than the area increase of ECs, which suggests that the ECs have become taller. Such morphological changes would decrease the vessel diameter, resulting in altered blood flow. The [Ca^2+^]_i_ of the astrocytes plays an important role in glial control of brain microcirculation [17], but our study also demonstrates that NH_4_Cl influences EC morphology directly, which could also contribute to the vasogenic effect of HA. However, the changes of EC area could also have an important influence on BBB permeability. We also demonstrated shrinkage of EC following acute removal of NH_4_Cl, which could be an important mechanism in the treatment of HE. It has been shown that the BBB permeability remains unchanged during hyperammonemia [23]. This is in agreement with our data showing unchanged astrocyte morphology and an increase in endothelial cell volume at a relatively small change of its surface projected area, but the results must be interpreted with caution as hyperammonemia was only transient. Other authors have reported significant swelling of brain tissue slices exposed to ammonia for a prolonged time up to three day [34]. It is possible that this effect of the prolonged exposure to ammonia results from additional biochemical and pathophysiological mechanisms triggered by elevated ammonia levels in the brain [23].

## Conclusion

Acute addition of NH_4_Cl elicits a transient rise of intracellular Ca^2+^ concentration in astrocytes and ECs. This is consistent with the results of previous studies [13]. The acute removal of NH_4_Cl leads to a transient rise of intracellular Ca^2+^ in both cell types, and the observed increase of [Ca^2+^]_i_ is shown to be due to release of Ca^2+^ from cytoplasmic proteins. The present study also shows that the addition and removal of NH_4_Cl results in an increase and decrease respectively of EC volume. Such effects were, however, absent in astrocytes. Moreover, the removal of NH_4_Cl, which is the basis of modern treatment of HE, resulted in a decrease in EC volume despite an accompanying transient increase in [Ca^2+^]_i_. Further studies are needed to determine the connection between these events.

## Abbreviations

ATP, adenosine-5’-triphosphate; BBB, blood–brain barrier; [Ca^2+^]_i_, intracellular calcium concentration; ECs, endothelial cells; [H^+^]_i_, intracellular H^+^ concentration; HA, hyperammonemia; HE, hepatic encephalopathy; pH_i_, intracellular pH; SBS – standard bathing solution.
